# Altered expression of transforming growth factor beta 1 and matrix metalloproteinase-9 results in elevated intraocular pressure in mice

**Published:** 2013-03-21

**Authors:** Jennifer V. Robertson, Anuja Siwakoti, Judith A. West-Mays

**Affiliations:** Department of Pathology and Molecular Medicine, McMaster University Health Science Centre, Rm 1R10, 1200 Main St. West. Hamilton, ON, Canada

## Abstract

**Purpose:**

Extracellular matrix remodeling is thought to have profound effects on tissue architecture and associated function. We have shown previously that overexpression of transforming growth factor beta (TGFβ), which stimulates matrix accumulation, results in altered morphology, cataract, and ocular hypertension in rodents. We have further shown that TGFβ-induced cataracts can be mitigated through inhibition of the matrix metalloproteinases (MMP) MMP-2 and MMP-9. We therefore sought to determine whether loss of MMP expression also altered TGFβ-induced changes in intraocular pressure (IOP).

**Methods:**

To carry out this study, TGFβ1 transgenic mice were bred onto a MMP-9 null background. IOP measurements were made at 1- to 2-, 2- to 3-, and 3- to 4-month time points using a TonoLab rebound tonometer. Histological and immunofluorescence findings were obtained at the same time points.

**Results:**

Our results demonstrate that lens-specific expression of TGFβ1 in mice results in altered morphology of the anterior segment and an accompanying significant increase in IOP. TGFβ1 transgenic mice bred onto the MMP-9 null background exhibited a further increase in IOP. Interestingly, the MMP-9-deficient animals (without the TGFβ transgene), which exhibited normal angle morphology, had increased IOP levels compared to their wild-type littermates.

**Conclusion:**

These results indicate that TGFβ and MMP-9 likely act independently in regulating IOP. Additionally, MMP-9 plays an important role in maintaining IOP, and further investigation into the mechanisms of MMP-9 activity in the anterior angle may give clues to how extracellular matrix remodeling participates in ocular hypertension and glaucoma.

## Introduction

Glaucoma is characterized by cupping of the optic nerve head and loss of retinal ganglion cells and is a leading cause of irreversible blindness, affecting approximately 70 million people worldwide [1,2]. Although the endpoint pathology in glaucoma occurs as damage to the retinal ganglion cells and optic nerve, glaucoma typically involves tissues in the front of the eye. Ocular fluid is produced by the ciliary body and flows from front part of the eye, around the iris, and drains out through the outflow pathway. The main aqueous outflow pathway of the eye consists of a series of endothelial cell-lined channels in the angle of the anterior chamber: the trabecular meshwork (TM), Schlemm's canal, the collector channels, and the episcleral venous system [3]. Disease often begins with a defect in this pathway, leading to reduced outflow facility, a subsequent rise in intraocular pressure (IOP), and followed by damage to the optic nerve. Changes in outflow facility can be due to physical closure of the angle (closed angle glaucoma) or as a result of malfunction without obvious anatomical changes (open angle glaucoma).

It has been hypothesized that in the case of open angle glaucoma, the pathological changes leading to aqueous outflow deficiency are the result of accumulated damage to the TM and Schlemm’s canal [4] that can involve chronic scarring and fibrosis of the TM. A known mediator of fibrosis, transforming growth factor beta (TGFβ), is present in the aqueous humor [5,6] and TGFβ levels have been elevated in patients with glaucoma [7-9]. In vitro studies involving perfused anterior segments [10,11] have also shown that following treatment with TGFβ2, outflow facility was decreased, IOP was increased, and expression of the extracellular matrix (ECM)–related genes plasminogen activator inhibitor-1 and fibronectin was increased. TGFβ can also cause significant alterations in the normal anatomic architecture of the anterior segment [12-14]. For example, as our laboratory has shown, adenoviral gene transfer of TGFβ1 to the anterior segment of the rat eye results in ocular hypertension, accompanied by aberrant proliferation and migration of corneal endothelial cells and iridocorneal adhesions, reminiscent of peripheral anterior synechiae observed in human patients with closed angle glaucoma [15]. TGFβ has also been shown to modulate the expression of proteins involved in the turnover of the ECM in the TM, in particular the matrix metalloproteinases (MMPs), which are known to influence outflow resistance [16].

MMPs, including MMP-2 and -9 among others, are elevated in the aqueous humor [17-19], optic nerve head [20,21], and chamber angle [19] of patients with glaucoma. Indeed, patients (and animals) undergoing trabeculotomy and filtering bleb [22-24] surgery also have increased levels of TGFβ and MMPs, suggesting that these molecules play a role in normal homeostatic responses in the eye. During normal wound healing, the ECM, both damaged and newly deposited, is remodeled by MMPs. When matrix remodeling becomes chronic, such as in the case of fibrosis, the levels of MMPs are often elevated, which may seem counterintuitive. However, elevated MMP activity is likely required to continually remodel the aberrant amount of matrix laid down during fibrosis. In fact, there is evidence that MMPs may enhance outflow facility in the short term [25]. With time, however, overexpression of MMPs can cause pathological tissue degradation and even fibrosis [26]. Moreover, since MMPs are now known to play additional roles, such as in activating cytokines and liberating growth factors from the matrix, MMPs may also function in stimulating the cascade of events involved in fibrosis of the outflow pathway, yet this is currently not well understood.

In this study, we used a transgenic mouse model that overexpresses TGFβ1 in the lens and aqueous humor [13] to initially determine whether the anatomical changes observed in the anterior segment of these mice results in elevated IOP. Transgenic expression of TGFβ1 resulted in altered anterior segment morphology and an accompanying increase in IOP. Since increased MMP expression, and specifically the gelatinase MMP-9, is commonly associated with TGFβ-induced fibrosis, we bred the TGFβ1 transgenic mice onto an MMP-9 null background to determine the role of MMP-9 in the initiation and progression of the glaucomatous features associated with elevated levels of TGFβ. Surprisingly, TGFβ transgenic mice bred onto the MMP-9 null background exhibited a further increase in IOP. Interestingly, MMP-9 null mice, without the presence of the TGFβ1 transgene, also exhibited elevated IOP levels compared to their wild-type littermates. Unlike the TGFβ transgenic mice, the MMP-9 null mice did not exhibit any overt changes in anterior segment morphology. Thus, normal expression of MMP-9 may be required to maintain IOP.

## Methods

### Animal treatment

Mice overexpressing constitutively active TGFβ1 under the αA-crystallin promoter were generated previously [13]. Mice lacking MMP-9 were purchased from Jackson Laboratories (Bar Harbor, ME). These mice were purchased on an FVBn background and backcrossed onto C57BL/6 for more than ten generations. To create parental strains, TGFβ transgenics (on C57BL/6 background) were crossed with MMP-9 knockout animals (C57BL/6 background) to give TGFβ positive (+)/ MMP-9 heterozygotes (+/−).The experimental animals were then created by breeding TGFβ^+^ /MMP-9^+/−^ mice with TGFβ^-^ /MMP-9^+/−^ mice. Fifty percent of each litter were used as experimental animals, and 50% remained as breeders. All animals were treated in accordance with the guidelines of the Canadian Council on Animal Care and according to the Association for Research in Vision and Ophthalmology Statement for the Use of Animals in Ophthalmic and Vision Research. All animals were housed under specific pathogen-free conditions, and rodent laboratory food and water were provided ad libitum*.* All animal procedures were performed under inhalation anesthesia with isoflurane (MTC Pharmaceuticals, Cambridge, Canada). IOP readings were taken under isoflurane inhalation after one drop of 0.5% proparacaine solution (P4554; Oakville, Canada) was applied to each eye. All readings were taken at 1–2, 2–3, and 3–4 months of age and were performed at the same time of the day. Readings were acquired with the Tonolab rebound tonometer (Topcon Canada, Inc, Waterloo, Canada). Several studies [27-29] have examined the reliability of the TonoLab to give consistent readings across a range of experimentally altered IOPs [30]. An average of nine independent valid readings was performed for each animal. Animals were euthanized at 1–2, 2–3, and 3–4 months of age by cervical dislocation, and their eyes were enucleated. One-way analysis of variance (ANOVA) was used to determine statistically significant differences between groups.

### Histology

After fixation in 10% neutral buffered formalin for 48 h, tissues were embedded in paraffin by routine methods (sample size n=3 for each treatment and time point). Four-micrometer-thick mid-sagittal sections were cut and stained with hematoxylin and eosin to visualize general tissue architecture.

### Immunohistochemistry

Studies to fluorescently localize alpha smooth muscle actin employed a monoclonal antibody conjugated to fluorescein isothiocyanate (clone 1A4; Sigma-Aldrich, Oakville, Canada; 1:200). In addition, sections were stained with polyclonal antibodies to collagen IV (Cedarlane Laboratories, Hornby, Canada; 1:200) and N-cadherin (Santa Cruz Biotechnologies, Santa Cruz, CA; 1:200). Secondary antibodies included goat anti-rabbit rhodamine and goat anti-rabbit fluorescein isothiocyanate (Bioshop Canada, Burlington, Canada; 1:500). All immunohistochemistry was performed as follows: After dehydration in a graded series of xylene and ethanol, paraffin sections were washed three times for 5 min with PBS (Sigma Aldrich, St Louis, MO; pH 7.3), boiled for 20 min in 10 mM sodium citrate (pH 6.09) for antigen retrieval, and then washed an additional three times in PBS. Sections were then blocked with a solution of 5% normal goat serum in PBS for 1 h at room temperature. After three washes of 5 min in PBS, the primary antibody was added to the sections in a volume of 100 µl and allowed to incubate overnight at 4 °C. After incubation, the sections were then washed three times for 5 min in PBS, and 100 µl of secondary antibody solution was added. The sections were incubated for 1 h at room temperature. After incubation, the sections were then washed three times for 5 min in PBS and then coverslipped with Vectashield mounting medium with 4', 6-diamidino-2-phenylindole (DAPI) as a nuclear counterstain (Vector Laboratories, Burlingame, CA). A sample size (n) of three was used for each treatment and time point.

### Microscopy

Fluorescently labeled sections were visualized using a Leica DMRA2 fluorescence microscope (Leica Microsystems Canada Inc., Richmond Hill, Canada) fitted with a Q-Imaging RETIGA 1300i FAST digital camera (Q-Imaging, Surrey, Canada). Images were captured using OpenLab software (PerkinElmer LAS, Shelton, CT). Cropping, rotating, and adding text to images were done using Adobe Photoshop 8 (Adobe Systems Canada, Ottawa, Canada).

## Results

**Figure 1:**
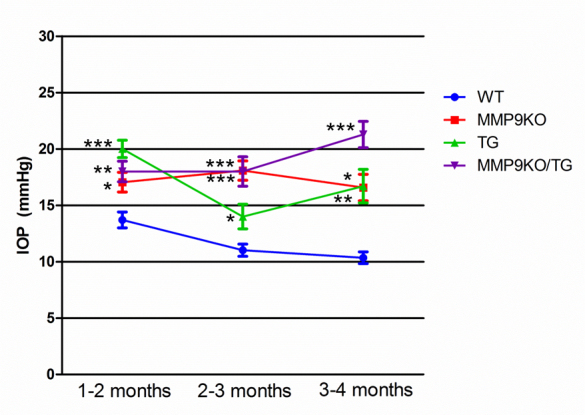
Intraocular pressure in mouse eyes over the course of 4 months. Data were obtained using the TonoLab under isoflurane anesthesia and represented as the mean intraocular pressure (IOP) in mm Hg. Error bars indicate the standard error of the mean (SEM). Transforming growth factor beta (TGFβ) transgenic (TG) mice, matrix metalloproteinase-9 knockout (MMP-9KO)/TGFβ1 transgenic mice, and MMP-9KO mice all exhibited elevated IOP compared to wild-type (WT) mice at all three time points examined. *** p<0.001; ** p<0.01 * p<0.05 (compared to WT).

Intraocular pressure was measured in mice that overexpress constitutively active TGFβ1 under the αA-crystallin promoter in the lens (TG) at 1–2, 2–3, and 3–4 months of age and compared to measurements taken from their wild-type littermates (WT; Figure 1). At 1–2 months of age, TG mice (n=27) demonstrated significantly higher IOP than WT mice (n=20; p<0.001). At 2–3 months of age, the IOP in the TG (n=10) and WT (n=13) animals had decreased somewhat. However, the IOP in the TG animals remained significantly higher (p<0.05) than in the WT group. At the 3- to 4-month time point, the IOP in the TG (n=10) group had increased again and remained significantly (p<0.01) higher than that in the WT (n=3) animals.

Mice expressing the TGFβ1 transgene (TG) were bred onto an MMP-9 null (KO) background to determine whether the loss of MMP-9 altered in any way the elevated IOP in this line of mice. At 1–2 months of age, the TGFβ transgenic/MMP9-KO (TG/MMP-9KO; n=17) mice exhibited IOP levels that were significantly higher than those of their WT (n=20) littermates (on the same mixed background; p<0.01; Figure 1). Importantly, the MMP-9 KO mice (n=16) without the TGFβ transgene also exhibited a significantly higher IOP than their WT littermates (p<0.05). The IOP levels in the MMP-9 KO and TG/MMP-9KO mice were not significantly different from each other at this age (p>0.05). At 2–3 months of age and 3–4 months of age, the MMP-9 KO (2–3 month [n=9]; 3–4 month [n=6]) and TG/MMP-9KO (2–3 month [n=10]; 3–4 month [n=17]) groups demonstrated significantly higher IOP compared to the WT mice (MMP-9KO, p<0.001 and p<0.05, respectively; TG/MMP-9KO, p<0.001 and p<0.001, respectively). Interestingly, the TG/MMP-9KO group of animals exhibited the highest IOP among all groups at 3–4 months of age. Together, these data suggest that loss of MMP-9 on its own results in higher IOP, and this increase is further increased with the TGFβ1 transgene.

**Figure 2:**
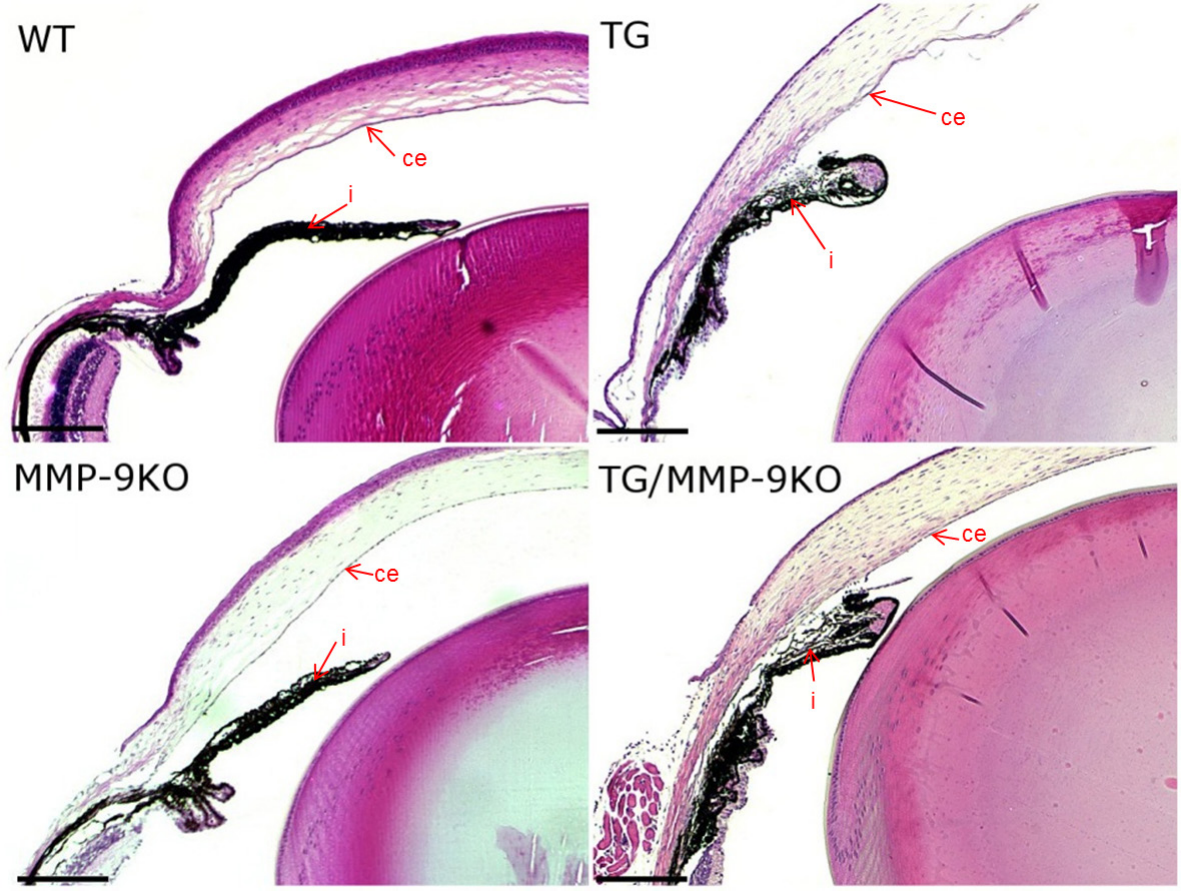
Hematoxylin and eosin–stained sections from 1- to 2-month-old mice. Adhesions between the iris (i) and corneal endothelium (ce) are easily visible in the transforming growth factor beta (TGFβ) transgenic (TG) and matrix metalloproteinase-9 knockout (MMP-9KO)/TGFβ1 transgenic mice but are absent in the wild-type (WT) and MMP-9KO mice. 5X, bar=200 µm.

**Figure 3:**
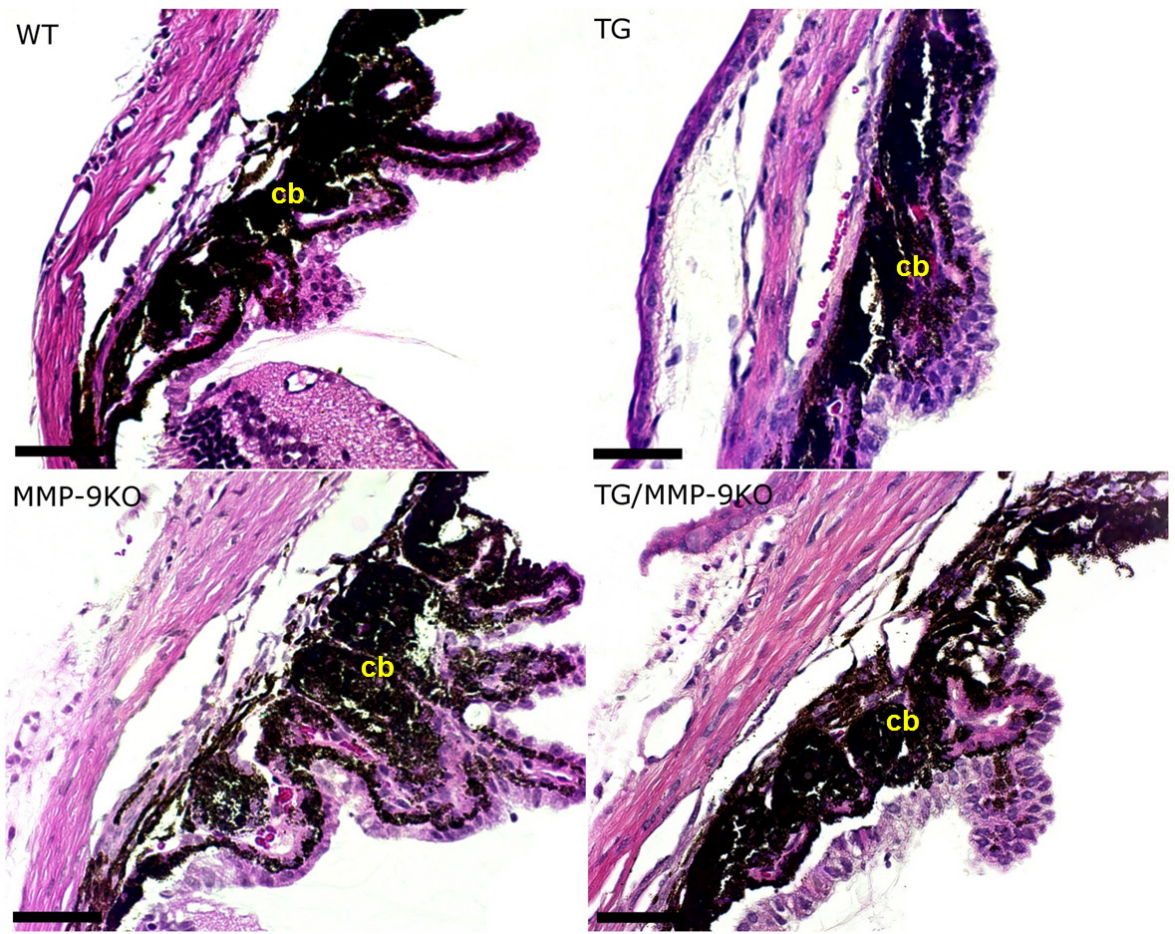
Hematoxylin and eosin–stained sections from 1- to 2-month-old mice. Altered development of the ciliary body (cb) is apparent in mice overexpressing transforming growth factor beta (TGFβ); however, these features were absent in wild-type (WT) and matrix metalloproteinase-9 knockout (MMP-9KO) mice. 20X, bar=100 µm.

**Figure 4:**
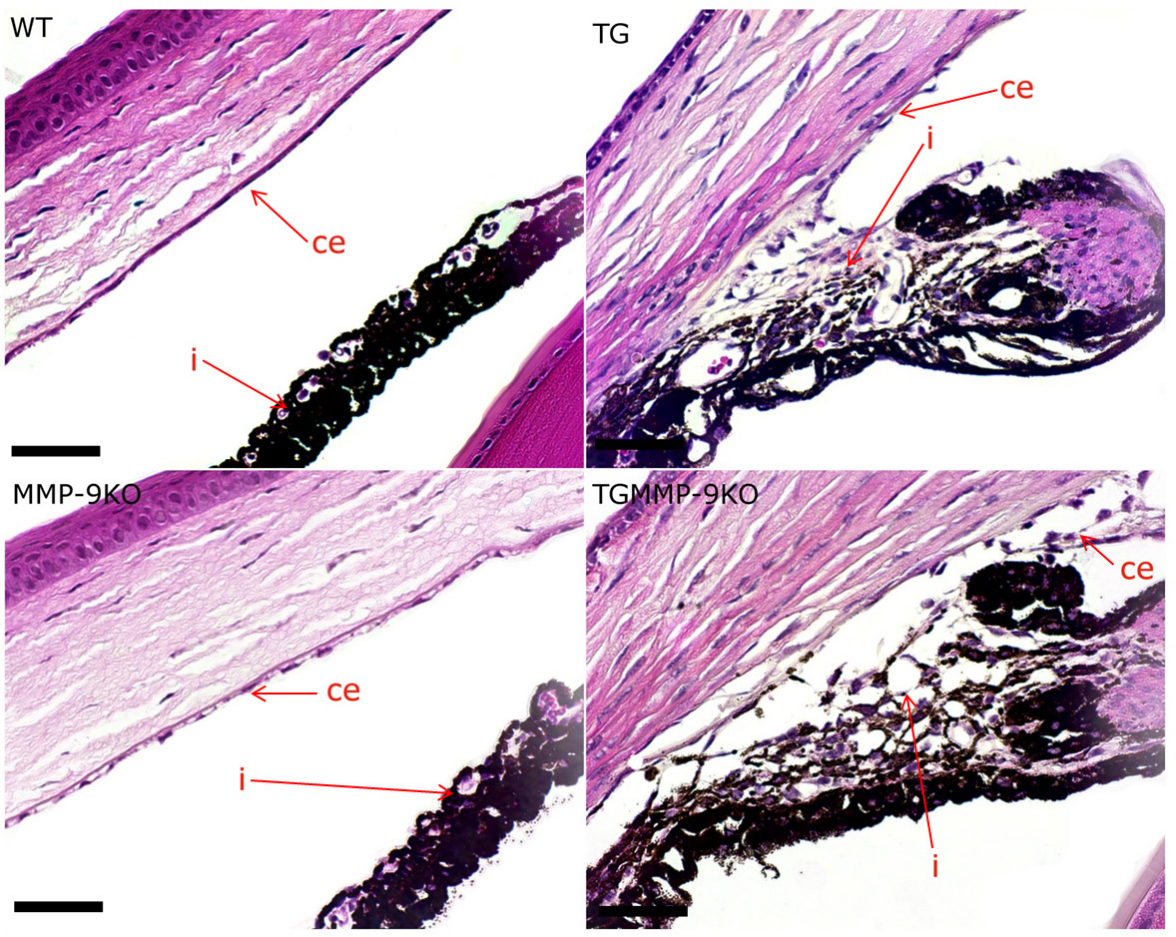
Hematoxylin and eosin–stained sections from 1- to 2-month-old mice. Adhesions seen in transforming growth factor beta (TGFβ) transgenic (TG) and matrix metalloproteinase-9 knockout (MMP-9KO)/TGFβ1 transgenic mice clearly demonstrate a change in the corneal endothelium (ce) and a hypercellularity of the iris (i) epithelium. These features were absent in the wild-type (WT) and MMP-9KO mice. 40X, bar=50 µm.

To determine morphological changes that may have led to the higher IOP observed in the TG and MMP-9KO mice, anterior segment morphology was examined using routine histological methods (Figure 2). At 1–2 months of age, all mice with the TGFβ1 transgene, the TG and TG/MMP-9KO groups, exhibited thicker corneas than the WT mice. The ciliary bodies in the TG and TG/MMP-9KO groups also appeared underdeveloped. Closer examination of the ciliary body (Figure 3) revealed that the pigmented and nonpigmented ciliary epithelial layers were present, and the lack of ciliary folds appeared concomitant with a striking deficiency in the ciliary process stroma. Additionally, the pars plana appeared to be lengthened (not shown), which was likely due to the lack of ciliary folds. These features were not evident in the WT mice or the MMP-9 KO mice, mice that did not express the TGFβ1 transgene. In addition, iridocorneal adhesions were evident and appeared to involve only the iris stromal layer as evidenced by the hyperproliferation of cells in this area, a feature absent in the anterior and posterior pigmented epithelia (Figure 4). Similar histological findings were found for the 3- to 4-month-old mice (data not shown).

**Figure 5:**
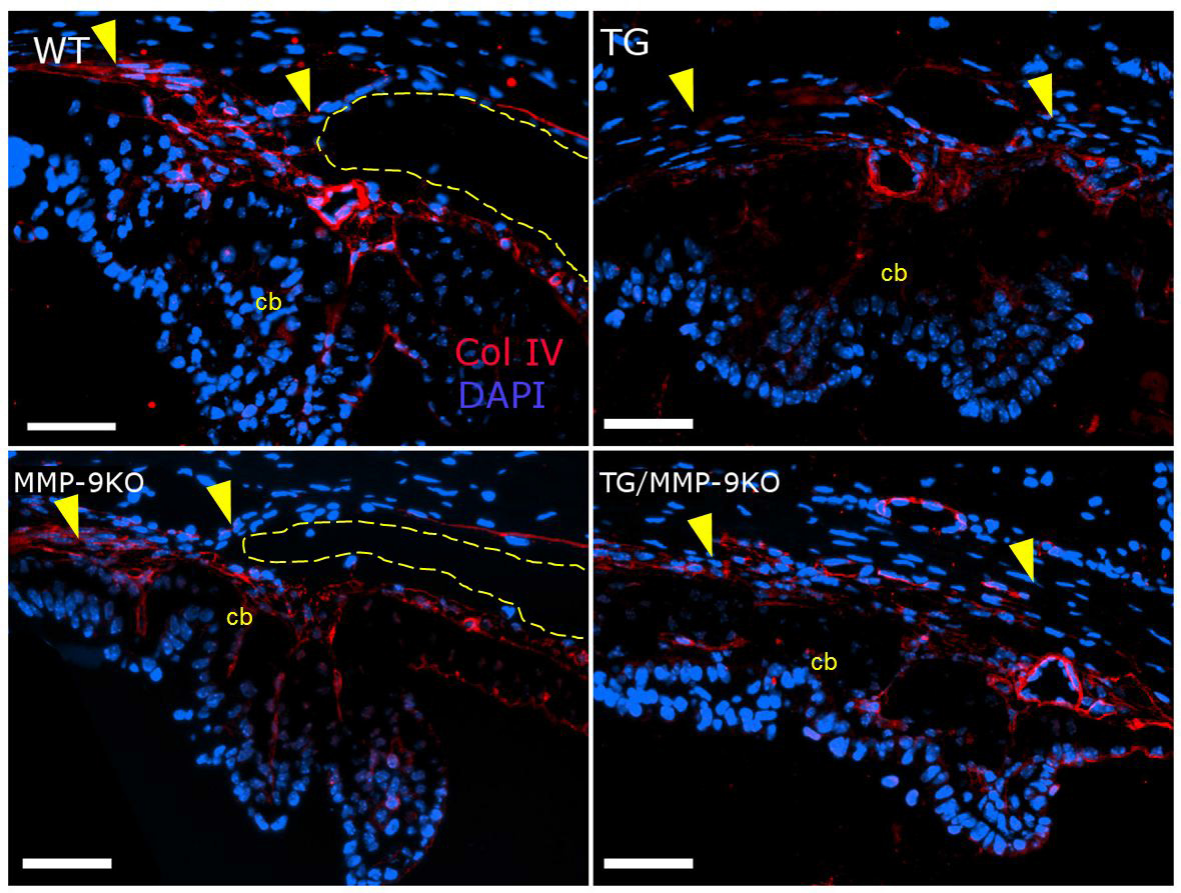
Immunolocalization of collagen IV (ColIV) in the ciliary body and angle of 1- to 2-month-old mice. Organized expression of ColIV can be seen in the trabecular meshwork (tm; region designated by the yellow arrowheads) and throughout the rays of the ciliary body (cb) in the open angles (yellow dashed line) of the wild-type (WT) and matrix metalloproteinase-9 knockout (MMP-9KO) mice. In the transgenic (TG) and TG/MMP-9KO mice, ColIV expression appears less organized. 40X, bar=50 µm.

**Figure 6:**
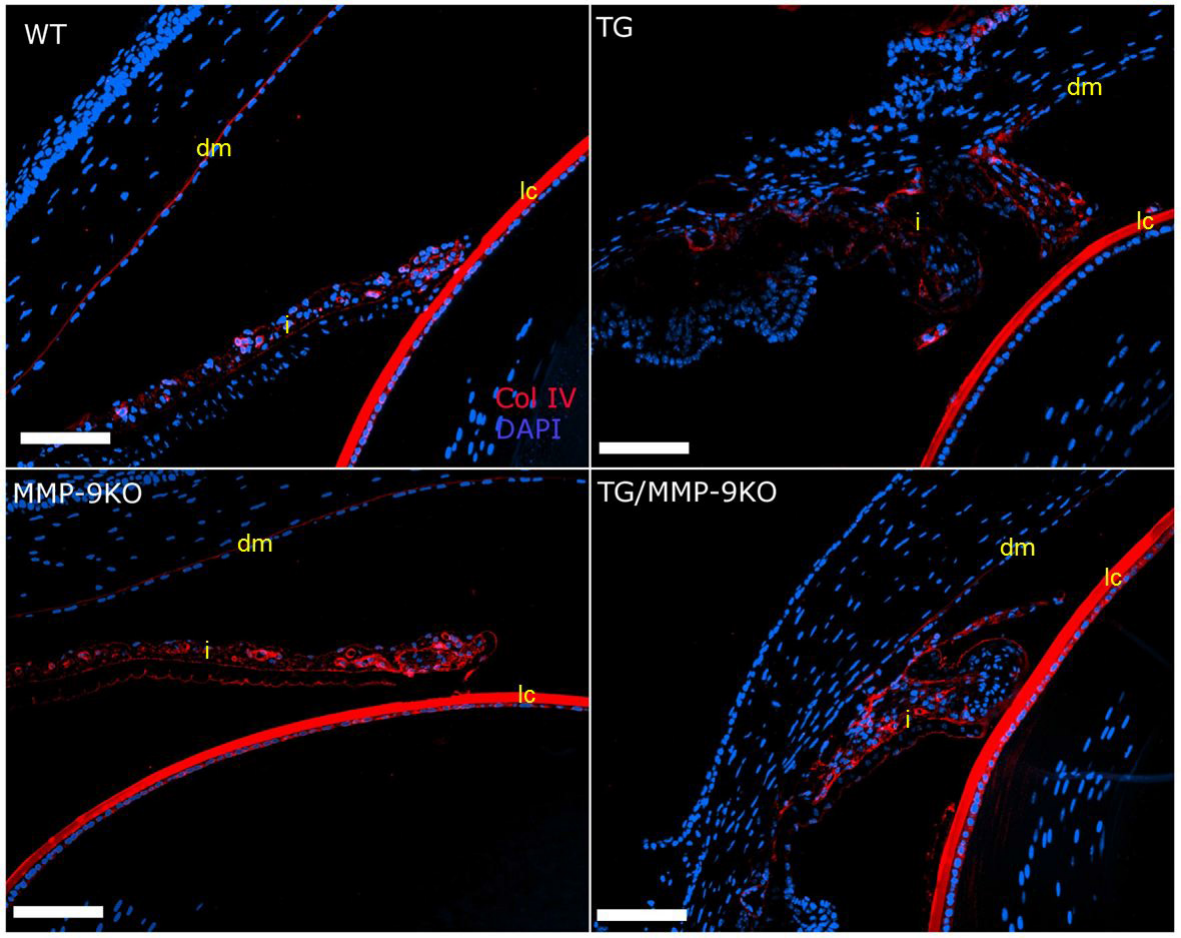
Immunolocalization of collagen IV (ColIV) in the iris of 1- to 2-month-old mice. Positive expression can be seen in the lens capsule (lc) in all groups of mice. Expression in the iris (i) and Descemet’s membrane (dm) is organized in the wild-type (WT) and matrix metalloproteinase-9 knockout (MMP-9KO) mice. Expression is also apparent in the transgenic (TG) and TG/MMP-9KO mice. However, it is distorted, consistent with the tissue distortion in these animals. 20X, bar=100 µm.

**Figure 7:**
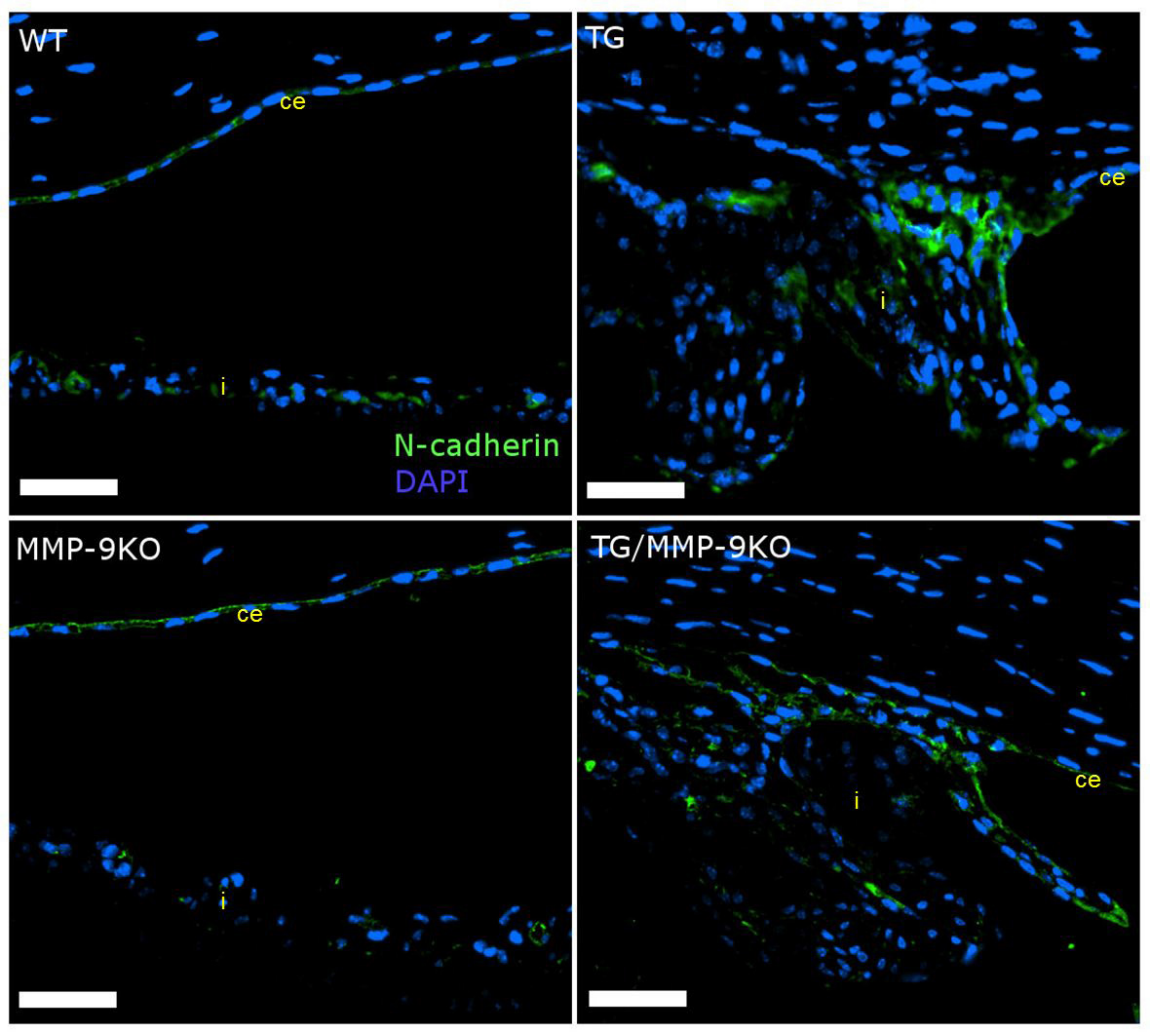
Immunolocalization of N-cadherin in eyes of 1- to 2-month-old mice. Expression of N-cadherin is detectable in the corneal endothelium (ce) and the iris (i) of wild-type (WT) and matrix metalloproteinase-9 knockout (MMP-9KO) mice. Increased expression is seen in the adhesion between the corneal endothelium and the iris epithelium in the transgenic (TG) and TG/MMP-9KO mice. 40X, bar=50 µm.

**Figure 8:**
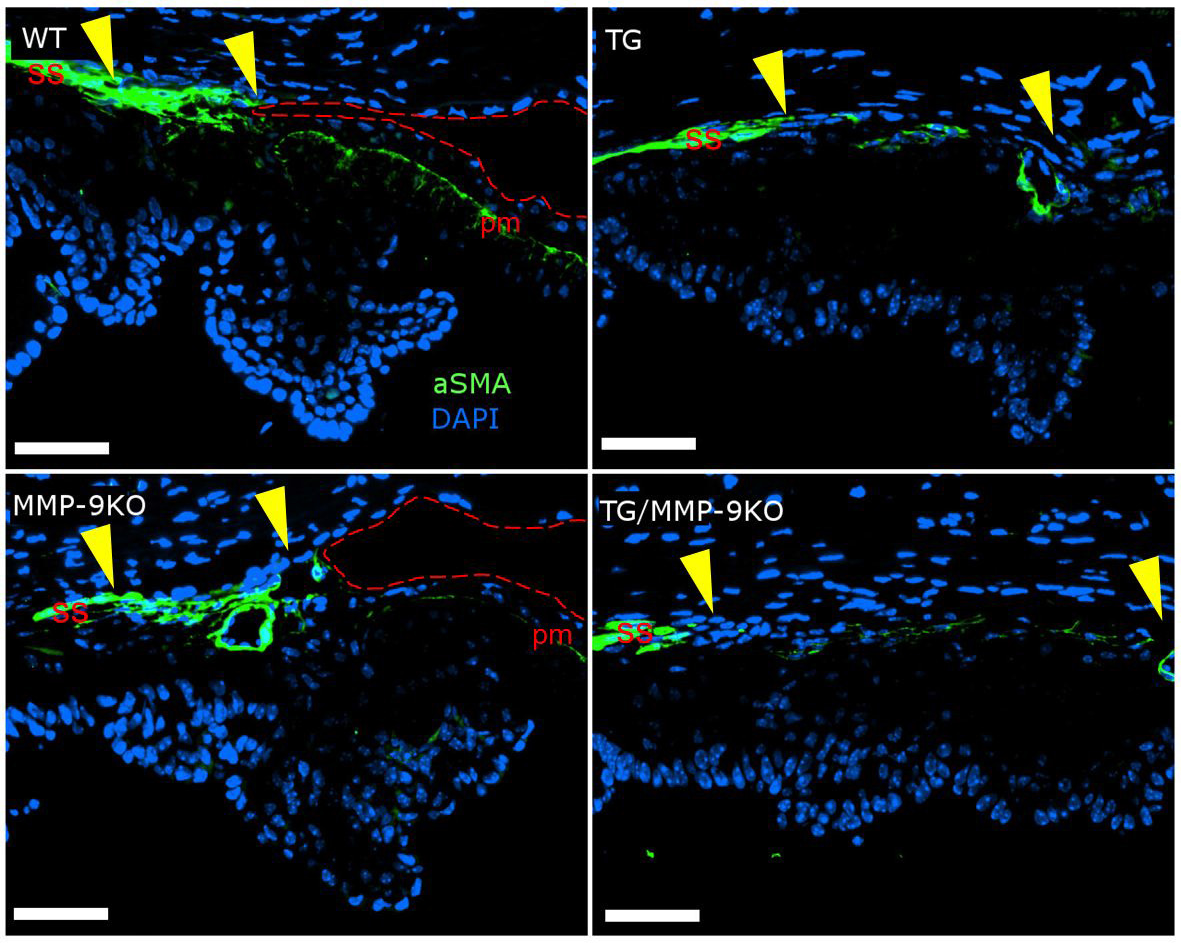
Immunolocalization of alpha smooth muscle actin in anterior chamber angles of 1- to 2-month-old mice. Expression of alpha smooth muscle actin (αSMA) is normal in wild-type (WT) mice, demonstrating a well-developed scleral spur (ss) and positive staining within the pupillary muscles (pm). Expression appears decreased in these areas in the matrix metalloproteinase-9 knockout (MMP-9KO) and transgenic (TG) mice. (The trabecular meshwork region is designated by the yellow arrowheads.) This decrease is exacerbated in the TG/MMP-9KO mice. An open chamber angle (red dashed line) can readily be seen in the WT and KO mice but not in TG and MMP-9KO/TG mice. 40X, bar=50 µm.

In a previous study, we found that administering adenoviral-delivered TGFβ1 (AdTGFβ1) to the anterior chamber of the rat eye resulted in anterior segment changes that were similar to those described for the TGFβ1 transgenic mice, including corneal thickening and iridocorneal adhesions [15]. Altered expression in various fibroproliferative markers was also discovered in the AdTGFβ1 rat model. Thus, we examined the expression of these proteins in the TG and MMP-9KO groups at 1–2 months (Figures 5, Figure 6, Figure 7, and Figure 8) and 3–4 months (data not shown) of age. In the TM, collagen IV expression was observed in parallel bundles extending into the fold of the ciliary body in the WT and MMP-9 KO animals (Figure 5). In contrast, the TG and TG/MMP-9KO groups (all examined) demonstrated disorganized expression of this molecule. In the iris and cornea (Figure 6), the WT animals showed normal distribution of collagen IV in the iris epithelia and surrounding the blood vessels. The MMP-9 KO animals appeared to demonstrate increased expression in this area. The TG and TG/MMP-9KO groups demonstrated abundant expression in the iridocorneal adhesions. Moreover, those animals in the TG/MMP-9KO group appeared to have increased collagen IV expression in this area compared to the TG animals. Similar findings were obtained for animals at 3–4 months of age (data not shown).

N-cadherin was next immunolocalized (Figure 7). The WT and MMP-9KO groups at 1–2 months of age showed typical expression of N-cadherin in the corneal endothelium, with minimal expression in the iris epithelium. In contrast, the TGFβ1 transgenic groups examined, TG and TG/MMP-9KO, demonstrated abundant expression of N-cadherin in the hypercellular region within the iridocorneal adhesions, similar to what we observed in the past in the AdTGFβ1-treated rats. Similar findings were obtained for the animals at 3–4 months of age (data not shown).

**Figure 9:**
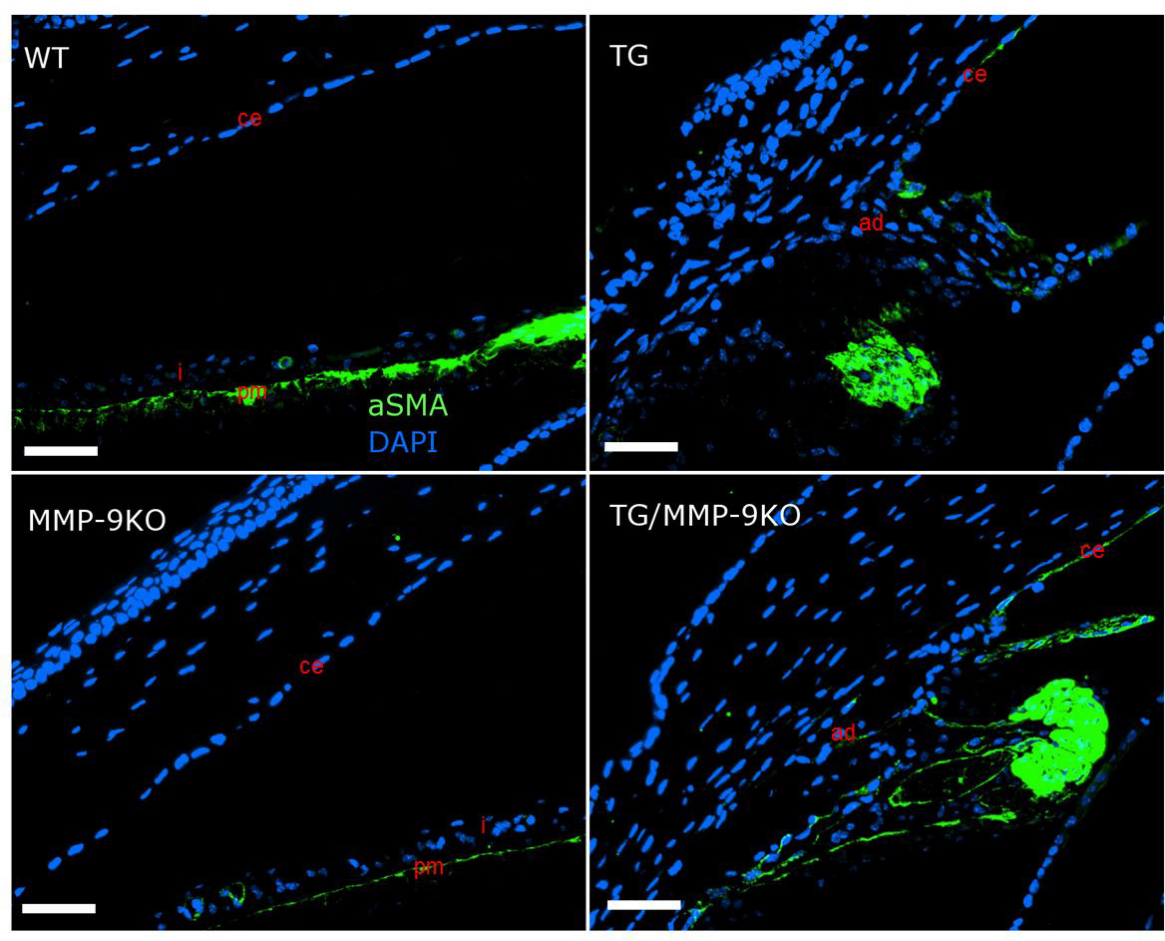
Immunolocalization of alpha smooth muscle actin in the iris and cornea of 1- to 2-month-old mice. The pupillary muscle (pm) is positively stained and easily discerned in the wild-type (WT) and matrix metalloproteinase-9 knockout (MMP-9KO) mice. The altered structure of the iris and adhesions (ad) between the anterior iris and the corneal endothelium resulted in aberrant expression of alpha smooth muscle actin (αSMA) in the transgenic (TG) and TG/MMP-9KO mice. 40X, bar=50 µm.

All of the groups at 1–2 months of age showed typical expression of alpha smooth muscle actin (αSMA) in the pupillary dilator muscle of the iris (Figure 8). However, in all of the TG and TG/MMP-9KO groups, αSMA expression was also observed in the area of the iridocorneal adhesions. In the TM, the WT animals demonstrated typical, abundant expression of αSMA (Figure 9). However, the TG and MMP-9 KO animals showed somewhat less αSMA expression in this area, with animals in the TG/MMP-9KO group exhibiting the least expression of αSMA in the TM region. Similar findings were obtained for animals at 3–4 months of age (data not shown).

## Discussion

Maintaining IOP is critical for retinal health and normal vision. Discovery of the mechanisms that contribute to ocular hypertension is therefore critical for developing effective preventative and/or therapeutic treatments. In this study, we examined the contributions of TGFβ1 and MMP-9, two genes known to regulate the dynamics of the ECM and suspected of controlling aqueous outflow. Initially, we examined the effect of TGFβ1 overexpression on IOP using a previously developed transgenic mouse line in which active TGFβ1 is chronically expressed from the lens, under control of the αA-crystallin promoter [13]. As reported previously, we found that these mice exhibited several defects in the anterior segment of the eye, including anterior subcapsular cataracts and iridocorneal adhesions resembling peripheral anterior synechiae formation in humans [13]. Importantly, we demonstrated that along with the dysmorphic changes in the anterior segment, the TGFβ1 transgenic mice exhibited significantly higher IOP than that of their wild-type littermates. The IOP levels in the TGFβ1 transgenic mice exhibited a decrease at the 2–3 month time point. However, this decrease was also observed in their wild-type littermates, and likely reflected a change in eye size in the mice as they were developing with age. The increased IOP in the TGFβ1 transgenic mice concurs with results of a previous study from our laboratory involving AdTGFβ1 to the anterior chamber of the rat eye in which similar changes in anterior segment morphology were observed and an accompanying increase in IOP [15]. Interestingly, a study using intracameral delivery of active AdTGFβ2 in rats also reported an induction in ocular hypertension [31]. However, these rats did not exhibit the anterior segment changes reported for the AdTGFβ1-injected rats. The authors further demonstrated that delivery of AdTGFβ2 reduced aqueous humor outflow facility in mice. Together, these findings indicate that overexpression of these TGFβ isoforms (in active form) can induce changes that resemble open and closed angle forms of glaucoma, and this results in elevated IOP.

The TGFβ1 transgenic mice, as well as those bred onto the MMP-9 null background (TG/MMP-9KO), also exhibited thickened corneas. Previous detailed investigation of the corneal phenotype of lens-specific TGFβ1 transgenic mice demonstrated that the change in corneal thickness is due to an increase in the thickness of the corneal stroma [13]. Thickened corneas were also reported for rats injected with AdTGFβ1 [15] and AdTGFβ2 [31]. To address whether a thickened cornea affects tonometry readings, investigations were performed by Shepard and colleagues in which IOP readings obtained from cannulation were compared with those taken using the tonometer [31]. The findings from these studies revealed that the tonometer-generated readings correlated well with the actual IOP readings in uninjected eyes and eyes injected with active AdTGFβ2. Thus, although the animals overexpressing TGFβ have thickened corneas, this is not likely to impact the accuracy of the tonometer readings [31].

In addition to the gross changes in the anterior chamber observed in the TGFβ1 transgenic mice, reduced expression of αSMA was observed in the TM compared to the wild-type littermates. This finding agrees with the decreased expression of αSMA previously reported by our laboratory in the TM of AdTGFβ1-treated rat eyes [15]. αSMA, a contractile protein, is expressed in the normal TM of humans and other species [32-39]. This suggests αSMA may play a role in modulating the outflow facility of the aqueous humor [40]. The fact that αSMA expression was decreased in the TM of TGFβ1 transgenic mice and AdTGFβ1-treated rats, both of which have accompanying ocular hypertension, supports this notion. Interestingly, in glaucomatous dogs, as ocular hypertension progresses, there is a loss of αSMA expression in the outer uveal and inner corneoscleral TM cells [32]. These in vivo results are, however, in contrast to several in vitro studies that show that TM cells, when exposed to TGFβ, express a higher level of αSMA [41]. Indeed TGFβ is a well-known regulator of αSMA [42]. As we have proposed previously [15], one possibility for the different findings may be related to the amount and duration of TGFβ elevation. For example, anterior segment tissues in vivo may show a different response to chronically elevated TGFβ compared to in vitro stimulation, which is often shorter. Additionally, the environmental cues in vitro are absent, which could alter the responsiveness of a given cell type to TGFβ stimulation.

Matrix metalloproteinases (MMPs) are elevated in the aqueous humor [17,18] and chamber angle [19] of patients with glaucoma, and MMPs are thought to control regulation of the aqueous outflow pathway. However, since MMPs have anti- and profibrotic functions, it was not clear what role MMPs may have in regulating IOP in vivo. Our laboratory has shown that MMP inhibition can suppress TGFβ-stimulated fibrosis in the lens. For example, using a TGFβ-induced model of anterior subcapsular cataracts in rats, we demonstrated that cotreatment with the MMP-2/9 specific inhibitor effectively prevented anterior subcapsular cataract formation and the associated deposition of matrix [43]. In addition, MMP inhibition has also been shown to prevent matrix deposition in injury-induced tissue remodeling [44,45]. We therefore hypothesized that MMP, and in particular MMP-9, deficiency, may attenuate the ocular phenotype and IOP levels observed in the TGFβ1 transgenic mice. We found the contrary; the TGFβ1 transgenic mice bred onto the MMP-9 null background exhibited the same fibrotic changes in the anterior segment as the TGFβ1 mice on the MMP-9 wild-type background. These findings demonstrate that MMP-9 does not play a role in mediating the fibroproliferative response in the anterior chamber of TGFβ1 transgenic mice.

Ocular hypertension was further elevated in the TG/MMP-9KO mice compared to the TG/MMP-9 WT mice. Furthermore, the MMP-9 KO mice, in the absence of the TGFβ1 transgene, exhibited increased IOP levels compared to their wild-type littermates. This occurred without the alterations in anterior segment morphology that were detected in the TGFβ1 transgenic mice. These findings suggest that a mechanism independent of that induced by TGFβ1 is responsible for the elevated IOP in the MMP-9 KO mice. MMPs have been shown, in human eye perfusion models, to play a role in regulating trabecular outflow, which directly impacts IOP [46,47]. For example, increasing activity of MMP-2, MMP-9, and MMP-3 in culture media of anterior segment tissue resulted in increasing the outflow rate by 160% [47]. Furthermore, inhibiting MMP activity significantly reduced the outflow rate [47]. These findings suggest that MMPs participate in ECM turnover in the TM, and when their activity levels are reduced, ECM accumulates, impeding outflow. However, demonstration of this mechanism in vivo and in glaucomatous eyes has not been reported. We did not observe any overt changes in ECM deposition in the TM of the MMP-9 KO mice. The mouse and human TM have similar structures. However, the TM in the mouse is much reduced in size compared to that in humans. Thus, detailed ultrastructural studies are required to rule out subtle changes in matrix deposition that may affect outflow. Finally, although MMPs are principally known for their ability to remodel the ECM, they have also been shown to participate in numerous signaling events, mediated by the cleaving of cytokine receptors or cell adhesion molecules and the activation of other MMPs. Thus, the loss of MMP-9 expression in the TM may alter cell signaling and thereby affect outflow and IOP.

In conclusion, we have demonstrated that alterations in expression levels of the extracellular matrix remodeling molecules, TGFβ and MMP-9, can impact the regulation of IOP. In particular, the loss of MMP-9 expression resulted in increased IOP levels, in the absence of any overt morphological changes. Further detailed analysis of the TM structure and aqueous outflow facility of the MMP-9 KO mice will help to further understand the how this enzyme contributes to regulating IOP.
